# MÖNCH detector enables fast and low-dose free-propagation phase-contrast computed tomography of *in situ* mouse lungs

**DOI:** 10.1107/S160057751701668X

**Published:** 2018-02-06

**Authors:** Christian Dullin, Jonas Albers, Giuliana Tromba, Marie Andrä, Marco Ramilli, Anna Bergamaschi

**Affiliations:** a Institute for Diagnostic and Interventional Radiology, University Medical Center, Robert Koch Strasse 40, Göttingen, Lower Saxony 37075, Germany; b Elettra-Sincrotrone Trieste, Strada Statale 14, km 163.5 in AREA Science Park, Trieste, Friuli Venezia Giulia 34149, Italy; c Paul Scherrer Institute, 5232 Villigen PSI, Switzerland

**Keywords:** mouse-lung imaging, low dose phase-contrast computed tomography, charge-integration detector

## Abstract

Due to the complexity of the underlying pathomechanism, *in vivo* mouse lung-disease models continue to be of great importance in preclinical respiratory research. Longitudinal studies following the cause of a disease or evaluating treatment efficacy are of particular interest but challenging due to the small size of the mouse lung and the fast breathing rate. Synchrotron-based in-line phase-contrast computed tomography imaging has been successfully applied in lung research in various applications, but mostly at dose levels that forbid longitudinal *in vivo* studies. Here, the novel charge-integrating hybrid detector MÖNCH is presented, which enables imaging of mouse lungs at a pixel size of 25 µm, in less than 10 s and with an entrance dose of about 70 mGy, which therefore will allow longitudinal lung disease studies to be performed in mouse models.

## Abstract 2

The high detective quantum efficiency of the novel charge-integrating hybrid detector MÖNCH enables *in situ* mouse lung imaging at a pixel size of 25 µm in 10 s with an entrance dose lower than 70 mGy, thereby enabling longitudinal *in vivo* synchrotron free-propagation phase-contrast computed tomography lung studies in mice.

## 1. Introduction   

Preclinical research of lung diseases often uses *in vivo* small-animal models because the complex interaction of the immune system to date cannot reliably be modelled with *in vitro* or *in silico* techniques (Fröhlich & Salar-Behzadi, 2014 ▸). Most of the models are realised in mice, which, due to their small size and fast breathing frequency, are especially challenging for imaging. Virtually only X-ray-based methods can provide the required spatial and temporal resolution to address anatomical alterations within the mouse lung *in vivo*. However, usually this requires a high radiation dose. Since there are no dose limitations for small-animal studies, appropriate dose levels are heavily discussed. Detombe *et al.* (2013 ▸) found no evidence of X-ray-induced lung fibrosis and inflammation directly after irradiation once a week with an entrance dose of 0.84 Gy for six weeks. This may be mainly related to the fact that these symptoms need a longer time to develop, as seen by Williams *et al.* (2010 ▸). Therefore, it could be argued that, due to the short lifespan of mice and the shorter duration of experiments, dose-related effects are of no critical importance. In contrast, Casciati *et al.* demonstrated that cellular and molecular effects are induced in the mouse brain by a low radiation dose of 100 mGy (Casciati *et al.*, 2016 ▸). Miyachi *et al.* state that mice irradiated with 500 mGy per day for six days showed increased analgesia (Miyachi, 1997 ▸). Miyahara *et al.* found no dose-related effects in resulting body weight gain, organ weight measurements, haematological test results, litter sizes, reared offspring sizes or histopathological findings comparing mice frequently scanned for four weeks with a total dose of about 200 mGy using *in vivo* micro-computed tomography with controls which were not scanned (Miyahara *et al.*, 2016 ▸). Moreover, studies show that mice have the same radiation sensitivity as humans and therefore we believe that mice need to be treated with the same caution (Back & Alexander, 1955 ▸). Overall, the reported data suggest that X-ray irradiation doses in the region of 1 Gy do not introduce deterministic effects immediately within mice, and mouse lungs in particular, and might therefore be tolerable for shorter experiments. Despite this, already at lower radiation doses, effects within the mice have been observed. It seems that imaging with a dose below 200 mGy can be considered harmless. However, reaching lower dose rates would still be preferable.

Propagation-based phase-contrast computed-tomography imaging (PBI) has already proven to provide images with a high contrast-to-noise ratio in soft-tissue applications (Fitzgerald, 2000 ▸). The benefit of PBI compared with classical absorption-based X-ray imaging is especially high in lungs, which provide a strong variation in the refractive index between soft tissue and air. Therefore, lung research continues to be one of the main applications of PBI (Yagi *et al.*, 1999 ▸; Kitchen *et al.*, 2005 ▸; Dullin, Larsson *et al.*, 2015 ▸). Lovric *et al.* (2013 ▸) demonstrated that PBI can be used for *in vivo* lung imaging at a resolution of 4–10 µm with a dose of 5–10 Gy providing highly detailed images of the alveolar structure of the mouse lung. However, the achieved dose seems to be too high for longitudinal *in vivo* mouse studies.

The use of monochromatic photon beams at synchrotron beamlines allows the energy of the photons to be chosen in order to optimize the contrast in the images while limiting the dose. However, one of the bottlenecks here is often the detectors which provide reduced detective quantum efficiency, since for pixels smaller than 50 µm they are normally based on phosphor conversion.

The MÖNCH detector developed by the detector group at the Paul Scherrer Institute (Dinapoli *et al.*, 2014 ▸; Ramilli *et al.*, 2017 ▸) is a charge integrating hybrid detector with a pixel size of 25 µm. Thanks to the direct conversion of X-rays in the silicon sensor and its low noise level, it provides single photon resolution, sufficient dynamic range, fast frame rate and simple operation at room temperature with a segmentation which could not be previously achieved for hybrid detectors.

Here we demonstrate that PBI in combination with the MÖNCH detector enables mouse-lung imaging with a resolution of 25 µm in a few seconds with an entrance dose of less than 70 mGy, allowing longitudinal *in vivo* mouse studies to monitor the cause of a disease and/or treatment response.

## 2. Methods   

### 2.1. *In situ* mouse lung samples   

In order to mimic *in vivo* conditions, two six-week-old female C57BL6 mice were sacrificed, a tracheotomy was performed, and the lungs were filled with air at a constant pressure of 30 cm water column. Following that, the trachea was tied and the entire mouse was embedded in 1% agarose gel in a 50 ml plastic tube as described by Dullin, dal Monego *et al.* (2015 ▸). No fixatives were used that may enter the air-filled spaces and change the refractive properties.

### 2.2. MÖNCH detector   

MÖNCH is a hybrid pixel detector with charge-integrating architecture and a pixel pitch of 25 µm (Dinapoli *et al.*, 2014 ▸).

The detector consists of a 320 µm-thick silicon sensor, with a detection efficiency of about 20% for 22 keV photons, bump-bonded with a 25 µm pitch to fully independent pixels in the readout chip. The MÖNCH 0.3 prototype used for this experiment consists of an array of 400 × 400 pixels, for a total area of 1 cm × 1 cm (Ramilli *et al.*, 2017 ▸). The whole matrix can be read out at a rate faster than 2000 frames s^−1^.

In this experiment, only the half of the detector area illuminated by the laminar synchrotron beam has been read out at a frame rate of 1000 frames s^−1^, with 10000 images acquired in a 10 s scan. The detector continuously streams data to the PC over a 10 Gb s^−1^ UDP interface, with a maximum data throughput of 10 Gb s^−1^ (320 kB/2)^−1^ = 7000 frames s^−1^ (10 Gbs from the interface, 320 kB from the size of one image, and the 2 due to the region of interest). A slower frame rate of 1000 frames s^−1^ has been selected for the experiment to limit the data throughput. Moreover, the images relative to 10 frames have been summed together to form a single projection, in order to increase the statistics. Since the amplifying and the readout sections of each channel of the readout chip can be disconnected from each other (Ramilli *et al.*, 2017 ▸), the exposure, *i.e.* the integration of the signal, can occur in parallel with the readout allowing a dead-time-free operation.

Compared with the performances of MÖNCH shown by Cartier *et al.* (2016 ▸), many photons are detected during each single image, and therefore the spectroscopic capability is lost and interpolation algorithms improving the spatial resolution beyond the pixel pitch cannot be applied. In this case, the point spread function (PSF) of the detector can be modelled as a trapezoid with 25 µm full width at half-maximum and 17 µm-wide slopes due to the charge-sharing contribution (Bergamaschi *et al.*, 2008 ▸, 2015 ▸). By comparing the standard deviation of the PSF with that of an ideal detector with 25 µm pixels, this would translate into a spatial resolution of about 30 µm.

To limit the dose, MÖNCH can use simultaneous exposure and readout, pushing the dead-time between frames to a few hundred nanoseconds, *i.e.* negligible compared with the 1 ms exposure time. However, in the experiment a dead-time of 85% has been erroneously configured, therefore effectively using only 15% of the dose delivered to the sample, although using simultaneous exposure and readout.

The dynamic range of the detector has been optimized by operating the preamplifier in high gain mode and by setting the gain of the correlated double sampling stage to 1 to measure the full flux provided by the beamline during the 1 ms exposure time, thus providing a saturation level of about 10 photons with an energy of 22 keV. Under these conditions, the noise has been estimated to be 74 ± 5 e^−^ RMS equivalent noise charge (ENC), which is defined as the noise in terms of the charge at the detector input needed to create the same output at the end of the analogue chain (Radeka, 1988 ▸). Taking into account the energy-to-charge conversion factor of 3.6 eV per electron–hole pair in silicon, the detector noise is about 265 eV, and the signal-to-noise ratio (SNR) at 22 keV is higher than 80, which gives the possibility of resolving single photons. The dynamic range of the detector could be still extended by a factor of five by reducing the gain of the preamplifier in order to support longer exposure times or higher photon fluxes. In this case, the electronic noise would increase to 215 ± 10 e^−^ RMS ENC, *i.e.* 775 eV, with a SNR at 22 keV greater than 28.

### 2.3. Image acquisition and phase retrieval   

Image acquisition was performed at the SYRMEP beamline of the Italian synchrotron light source Elettra, operated at 22 keV with a sample-to-detector distance of 1.5 m. A 180° scan with a rotation speed of 18° s^−1^ was performed resulting in a total scanning time of 10 s. Before the reconstruction, the 10000 acquired angular projections were averaged to a set of 1000 projections. A single distance phase-retrieval algorithm based on the transport of intensity equation and implemented in the SYRMEP tomo project reconstruction framework (STP) (Brun *et al.*, 2017 ▸) was used with a nominal ratio between real and imaginary part of the complex refractive index of 1950 (Mohammadi *et al.*, 2014 ▸). Data sets were then reconstructed using the standard filtered back-projection algorithm implemented in STP. For three-dimensional rendering, the software *SCRY* (version 6) (Kuchel & Sautter GbR) developed by the authors was used.

### 2.4. Dose measurement   

To measure the X-ray dose within the mouse, two thermo­luminescence detectors (TLDs) were implanted within the chest of an additional mouse *ex vivo*. TLDs (GR-200 A, based on LiF:Mg,Cu,P; Hangzhou Freq-control Electronics Technology Co., Ltd) of size 4.5 mm × 4.5 mm were used. The TLDs were wrapped in parafilm (Sigma Aldrich) to protect them from moisture. The TLD-bearing mice were exposed to radiation using the same parameter used for the lung computed tomography. An average dose of 48.8 ± 5.8 (mean ± std) was measured.

### 2.5. Ethical statement   

*Ex vivo* mouse samples were obtained from mice expelled from a breeding project. All animal procedures were performed in compliance with the guidelines of the European Directive (2010/63/EU) and Italian ethical laws, and were approved by the Italian Ministry of Health on 26 February 2016 (No. 23402.2).

## 3. Results and discussion   

**Figure 1:**
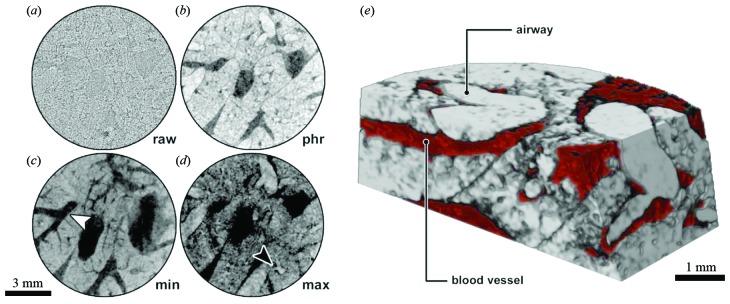
Imaging results for an *in situ* mouse lung: data acquired in 10 s, sample-to-detector distance 142 cm, *E* = 22 keV, pixel size = 25 µm. (*a*) Reconstructed raw data of a central slice through the lung (field of view ∼1 cm × 1 cm × 0.4 cm). A high noise level but also strong phase effects are visible. (*b*) The same slice reconstructed after single distance phase retrieval (delta-to-beta ratio of 1950). Lung septum, bigger air-filled spaces (bright), vessels and soft tissue (dark) are displayed. (*c*) The projected minimum over ten slices shows vessels (white arrowhead) and soft tissue. (*d*) The projected maximum over ten slices displays airways (black arrowhead). (*e*) Volume rendering representation, virtually cut to show airways (grey) and vessels and soft tissue (red).

In order to evaluate the potential of the MÖNCH detector for *in vivo* lung imaging in mice, local area scans of mice lungs *in situ*, inflated with air, were performed. The resulting image quality and the level of detail in which the mouse lung can be studied is depicted in Fig. 1 ▸ for one mouse exemplarily.

In Fig. 1(*a*) ▸, a slice of the reconstructed raw data is shown, demonstrating a high noise level but also the presence of phase effects. Fig. 1(*b*) ▸ depicts the same slice reconstructed after single distance phase retrieval was applied (Paganin *et al.*, 2002 ▸) using the nominal delta-to-beta ratio of 1950 for lung tissue (Mohammadi *et al.*, 2014 ▸). Bigger air-filled spaces, bronchi as well as the septum between the lung lobes are depicted. Interestingly, if the minimum of the grey values of ten consecutive slices is projected [Fig. 1(*c*) ▸], blood vessels, which due to their blood content show less phase shift than the air-filled spaces, can be visualized (white arrowhead). If instead the maximum of the grey values is projected, the bronchi are more pronounced (black arrowhead). Our method of preparation has markedly contributed to the successful discrimination of blood vessels and airways. Scanning the lung *in situ* reduces the risk of blood or other liquids entering the airways which could lead to a lower contrast and thus diminish the distinction between blood vessels and airways. Therefore, using phase-contrast computed tomography to analyse lungs *in situ* not only allows their natural shape to be maintained but also avoids additional alterations of the organ thereby providing more reliable readouts. The acquired and processed data allow for three-dimensional rendering of the imaged central part of the lung as demonstrated in Fig. 1(*e*) ▸. Volumes of air are coloured in grey and vessels and other soft tissue in red. The data are virtually cut to facilitate the display of the bronchi and a blood vessel.

Since the detector was not running dead-time-free, only about 15% of the measured 68 mGy entrance dose contributed to the image formation. This measured entrance dose is supported by the measured absorbed dose of about 50 mGy within the chest cavity of an additional mouse implanted with TLDs and exposed to X-rays using the same protocol. Therefore, the same image quality for *in situ* local area mouse-lung scan utilizing MÖNCH should be achievable with a dose of ∼10 mGy, thereby lowering the acquisition time from 10 s to 1.5 s.

In lung imaging, the motion of the chest leads to artefacts. Therefore, two methods are commonly employed in small-animal imaging: retrospective gating (Bartling *et al.*, 2007 ▸) and breath-hold imaging (Boll *et al.*, 2010 ▸). For the former, more than one set of angular projections needs to be acquired which can then be sorted for different expansion states of the chest, generating a subset that allows reconstruction of a motion-free three-dimensional image. The second method, breath-hold imaging, requires intubation of the animal and forced ventilation. During acquisition, the ventilation is blocked which stops the chest movement. It has been shown that breath hold times of 30 s up to 1 min are tolerated by mice. Each method has advantages and disadvantages: retrospective gating can work in combination with spontaneous breathing, but uses higher radiation doses. Breath-hold imaging is the more dose-efficient method but requires intubation, which, especially in mice, involves the risk of irritating or damaging the trachea which might influence the experiment. Due to the high detective quantum efficiency of the MÖNCH detector, both methods are applicable.

## 4. Conclusion   

It has been demonstrated that in-line free-propagation phase-contrast computed-tomography imaging of *in situ* mouse lungs can be achieved with a pixel size of 25 µm in 10 s with an entrance dose of less than 70 mGy utilizing the MÖNCH 0.3 detector developed at the Paul Scherrer Institute. This enables longitudinal *in vivo* lung studies in mouse models at a synchrotron light source.

In addition, since the detector was erroneously not used dead-time-free and only 15% of the entrance dose at the sample position contributed to image formation, and the rotation stage had a maximum angular speed of 18° s^−1^, the same image quality would be achievable with a dose of about 10 mGy in 1.5 s. The quantum efficiency could easily be increased to about 30–50% by using a 450–1000 µm-thick silicon sensor instead of one of 320 µm thickness. To date, the MÖNCH 0.3 prototype detector has a field of view of 1 cm × 1 cm, which limits the application to local area scans within the lung. A prototype with a field of view of 2 cm × 3 cm is planned, which in combination with a beam expander would allow the entire mouse lung to be imaged at 30 µm resolution in one rotation with an entrance dose of approximately 60 mGy.

Here it was demonstrated that, for moderate spatial resolution, the high detective quantum efficiency provided by direct conversion detectors gives clear advantages compared with higher absorption efficiency phosphor-based systems in terms of dose, still with reasonably short exposure times thanks to the fast frame rate and large dynamic range.
